# Relationship Between Body Composition and Quality of Life in Young Patients With Differentiated Thyroid Carcinoma After Thyroidectomy

**DOI:** 10.1155/ije/9268141

**Published:** 2026-05-22

**Authors:** Carla Tamanini Ferrari Schock, Lígia de Moraes Antunes-Correa, Fabiana Lascala Juliani, Marcelo Alcântara De Santana Souza, José Barreto Campello Carvalheira, Luís Bahamondes, Denise Engelbrecht Zantut-Wittmann

**Affiliations:** ^1^ Endocrinology Division, Department of Internal Medicine, School of Medical Sciences, University of Campinas, Campinas, São Paulo, Brazil, unicamp.br; ^2^ School of Physical Education, University of Campinas, Campinas, São Paulo, Brazil, unicamp.br; ^3^ Oncology Division, Department of Anesthesiology, Radiology and Oncology, School of Medical Sciences, University of Campinas, Campinas, São Paulo, Brazil, unicamp.br; ^4^ Department of Obstetrics and Gynecology, School of Medical Sciences, University of Campinas, Campinas, São Paulo, Brazil, unicamp.br

**Keywords:** body composition, differentiated thyroid carcinoma, hypothyroidism, quality of life, total thyroidectomy

## Abstract

**Objectives:**

To assess body composition (BC) and quality of life (QoL) in young patients with differentiated thyroid carcinoma (DTC) after total thyroidectomy.

**Methods:**

Fifty‐one patients with DTC were included. Clinical, demographic, and anthropometric data were collected. BC was assessed using dual‐energy X‐ray absorptiometry (DXA), QoL was assessed with the SF‐36 questionnaire, and DTC features and thyroid function parameters were analyzed.

**Results:**

Patients with DTC, with a mean age of 35 years and a mean BMI of 28.5 kg/m^2^, showed SF‐36 physical and mental component summary (PCS; MCS) scores slightly shorter than 50 (100 being the best) and higher total fat percentage (TFP), fat mass (FM), and fat mass index (FMI). A negative correlation was observed between FMI and FM with general condition (GC) as measured by the SF‐36. Higher free T4 and better GC were predictors of lower body fat. Older age, higher levothyroxine dose, and better GC predicted lower FMI. Age, levothyroxine dose, TSH, and higher PCS score were predictors of lower TFP. A higher GC score also predicted lower FM.

**Conclusions:**

Young thyroidectomized patients with DTC presented a compromised QoL and BC profile, with increased FM, TFP, FMI, and BMI. Alterations in BC were associated with worse QoL, suggesting a negative impact of DTC on patient well‐being. These findings emphasize the importance of a multidisciplinary management approach that considers both BC and QoL in patients with DTC under levothyroxine therapy, aiming to improve overall outcomes and QoL.

## 1. Introduction

Thyroid hormones (THs) are considered the main endocrine regulators of energy balance. In this sense, TH plays a central role in determining energy expenditure (EE), body mass index (BMI), and body composition (BC), including fat mass (FM), bone mass, and mainly fat‐free mass. It is well established that both hypo and hyperthyroidism significantly alter these processes, causing changes in the metabolic profile and in the BC [1].

It was described that levothyroxine (LT4) replacement therapy was associated with lower resting EE, although thyrotrophin (TSH) levels remained within the reference range. This would corroborate the fact that many treated patients with hypothyroidism complain of weight gain, despite TSH levels within the target [2]. One of the explanations could be reduced circulating levels of free triiodothyronine (T3). Approximately half of the individuals on LT4 replacement had free T3 at levels below the reference range, despite mean TSH values of 2.08 mIU/L. Low levels of T3 have been described in euthyroid individuals treated with LT4 (reviewed by Jonklaas et al.), with one study showing that TSH levels below the reference were necessary to restore T3 presurgery levels in patients after thyroidectomy. Therefore, an investigation of EE and the effects of T3 on hypothyroidism treated after thyroidectomy is necessary [3].

The relationship between quality of life (QoL) and BC and its influence in athyreotic individuals, even in the euthyroid state, is not well understood. Scarce studies included middle‐aged females, generating several confounding factors associated with weight gain and metabolic changes that occur due to energy balance positive, changes in metabolism, and BC associated with menopause.

In a cross‐sectional study with 6983 participants, 30.8% of women using LT4 alone had a poor QoL score in the domain of vitality, 30.5% in mental health, 25.1% in social functioning, 23.8% in bodily pain, and 37.5% in general health, compared to controls. There was no difference in cognitive functioning between women using LT4 and the control group. When evaluated in relation to the presence of the D2‐*Thr92Ala9* polymorphism, there was no difference between the parameters [4].

Regarding TSH levels and BC of patients undergoing postoperative follow‐up for thyroid cancer, there is scarce information, and it is a controversial issue in the literature. Some authors have not found differences in resting EE or BC of patients undergoing total thyroidectomy (TT) compared to controls, while others have found no change in TBM, but observed a 9% decrease in muscle mass in the group with differentiated thyroid carcinoma (DTC) after surgery. A third study which randomly evaluated patients with DTC assigned to continue LT4‐suppressive therapy versus reducing LT4 doses for 6 months found no changes in BC despite an increase in mean TSH levels of 0.07 at 4.35 mIU/L. These findings suggest that there is a variety in the “metabolic phenotype” of these patients, including the sex, quantitative and qualitative food intake, and physical activity levels, which would be more determinant in the corporeal changes in TSH levels within the reference range. Consequently, long‐term minimally suppressive doses of LT4 do not have major salutary or adverse effects on EE or BC [5].

Patients who have undergone TT often report a worsening of their QoL and changes in BC. Associated with the state of hypothyroidism, the concomitant occurrence of DTC should be highlighted, and the influence of this condition cannot be ruled out. To date, the literature is scarce in demonstrating a direct association between TT and these complaints. Therefore, the aim of our study was to analyze BC and QoL of young patients undergoing TT due to DTC.

## 2. Methods

### 2.1. Study Design

We conducted a cross‐sectional study at a specialized center for DTC. The study was approved by the university’s ethics committee (CAEE 66969522.4.0000.5404), and all participants provided written informed consent. We evaluated BC and QoL in athyreotic patients after TT for DTC, receiving LT4 replacement therapy. Clinical characteristics, BC, QoL, and TH profiles were analyzed. Patient recruitment occurred between January 2023 and January 2024. Eligible participants included men and women over 18 years of age with a confirmed diagnosis of DTC, who had complete clinical data, underwent dual‐energy X‐ray absorptiometry (DXA) assessment, underwent biochemical assessment of thyroid function, and completed the SF‐36 QoL questionnaire.

The clinical, demographic, and anthropometric data were assessed, include age, sex, weight (kg), height (cm), and BMI (kg/m^2^). We assessed life habits like smoking (yes > 100 cigarettes in life, ex‐smoker < 100 cigarettes in life, or never smoker), sun exposure (sufficient > 20 min a day and > 3 times a week or insufficient < 20 min a day and < 3 times a week), caffeine intake (less than 200 mg/day and greater than 200 mg/day), and physical activity which was defined as the practice of regular physical exercise. Participants were classified as *active* (aerobic physical activity > 150 min per week) or *inactive* (no aerobic physical activity or < 150 min per week). This variable was self‐reported and assessed at the time of evaluation. Dietary intake was not formally quantified, representing a potential confounding factor. Time since surgery was recorded and included in the analyses.

Data on cancer characteristics and treatment were collected, including total LT4 dose and dose adjusted per body weight (μg/kg), time since diagnosis, type of surgery (TT or TT with neck lymph node dissection), ablative and cumulative radioiodine activity, staging for DTC‐related mortality risk (TNM), initial risk stratification for persistence or recurrence according to the American Thyroid Association (ATA), and risk reclassification based on ATA response after 1 year of treatment. Tumor characteristics comprised histological type and subtype of thyroid carcinoma, presence of multifocality, vascular, capsular, or perineural invasion, extrathyroidal extension (microscopic or macroscopic), and whether radioiodine therapy (RIT) was performed. Laboratory data included serum concentrations of thyroglobulin (Tg), anti‐thyroglobulin antibody (TgAb), and thyroid function tests (TSH and free T4 [FT4]).

### 2.2. QoL—SF‐36 Questionnaire

The SF‐36/RAND‐36 was applied to measure health perception QoL in DTC patients. The instrument includes scales for physical and social assessment, limitations of function due to physical or emotional problems, mental health, energy, pain, and general perception of health. A high score corresponds to a better state of health. The SF‐36 was scored in two stages. The precoded numeric values were first recorded according to the scoring key in Step 1. Each item was rated on a scale of 0 (lowest) to 100 (highest). The eight scores of the scale were calculated in Step 2, using the average of the items within the same scale [6].

### 2.3. BC

BC was assessed at a single time point using total body densitometry with DXA (LUNAR DPX device, GE Healthcare Corporation, Madison, WI, USA). The following parameters were analyzed: total body mass (absolute body mass, equivalent to body weight, expressed in kilograms); FM (amount of body fat tissue, expressed in grams); total fat percentage (TFP, proportion of FM relative to total body mass); lean mass (LM, amount of lean tissue, expressed in grams); total lean mass/height^2^ (LM adjusted for height, expressed as kg/m^2^); fat mass index (FMI, FM in kilograms divided by height squared in meters); and Baumgartner index (appendicular LM relative to body surface area, used to identify low muscle mass and sarcopenia).

### 2.4. Biochemical Analyses

TSH—electrochemiluminescence immunoassay—reference value (RV): 0.41–4.5 mIU/L; FT4—electrochemiluminescence immunoassay—RV: 0.9–1.8 ng/dL; Tg RV < 0.2 ng/mL; TgAb RV < 115 UI/mL.

### 2.5. Statistical Analysis

To describe the sample profile according to the variables under study, frequency tables were created for categorical variables, displaying absolute frequency (*n*) and percentage (%) values, as well as position and dispersion measures for numerical variables. To correlate numerical variables, Spearman’s linear correlation coefficient was used. To analyze variables related to BC and the components of the SF‐36, multiple linear regression analysis with selective stepwise variable selection was used. The variables were transformed into positions (ranks) due to the absence of normal distribution. The significance level was 0.05%.

## 3. Results

The study cohort had a mean age of 35 years, with 70.6% of participants being female. Regarding BMI, 31.4% ≤ 25 kg/m^2^, 33.3% ranged from 25.1 to 30 kg/m^2^, 27.4% ranged from 30.1 to 34.3 kg/m^2^, and 7.8% ≥ 35 kg/m^2^. TH profile was in RVs (Table 1). Papillary thyroid carcinoma represented 96.1% of the diagnoses, while 3.9% corresponded to the follicular subtype. The mean time since thyroidectomy was 7 years. Regarding risk stratification, 44.9% were classified as intermediate initial ATA risk and 32.7% as low risk, whereas 52% achieved an excellent response to treatment. Further histopathological details, therapeutic strategies, clinical outcomes, and lifestyle characteristics are provided in the Supporting Material (Tables S1 and S2).

**TABLE 1:** Descriptive analysis of patients with differentiated thyroid carcinoma regarding demographic, anthropometric, and thyroid hormonal profile data.

Variable		Differentiated thyroid carcinoma *n* = 51
Age (years)		35.7 ± 7.7

Sex	Female	36 (70,6%)
	Male	15 (29.4%)

Weight (kg)		78.1 ± 18.7

Height (cm)		165.5 ± 9.6

Body mass index (kg/m^2^)		28.5 ± 5.6

TSH (mIU/L)		2.2 ± 7.6

Free T4 (ng/dL)		1.4 ± 0.3

*Note:* Continuous variables are expressed as mean + standard deviation. Categorical variables are expressed as absolute number (*n*) and frequency (%). TSH: thyrotrophin.

### 3.1. QoL (SF‐36)

**FIGURE 1:**
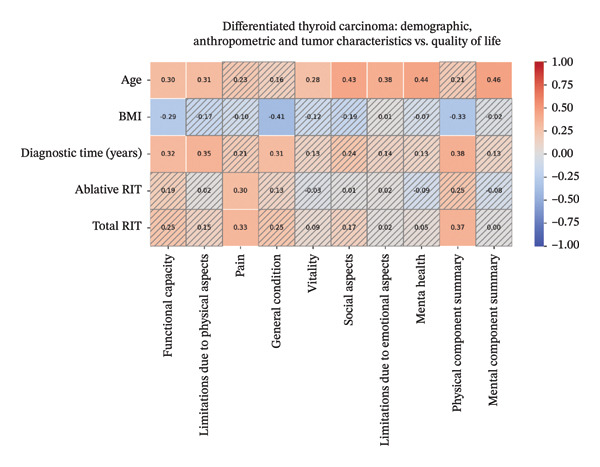
Spearman’s linear correlation heatmap demonstrates values of ῤ between demographic, anthropometric, and tumor characteristic variables with quality of life in patients with DTC. Values of *p* > 0.05 are not shaded.

SF‐36 QoL assessment demonstrated reduced scores across domains (Table 2). In DTC, there was a positive correlation between age and functional capacity (FC), limitations due to physical aspects (LP), vitality, social aspects (SA), limitations due to emotional aspects (LE), mental health, and mental component summary (MCS); between time of DTC diagnosis and FC, LP, general condition (GC), and physical component summary (PCS); between RIT ablative activity and pain; and between total radioiodine activity and pain and PCS. A negative correlation was found between BMI and FC, GC, and PCS (Figure 1, Supporting Material Table S3). Weight, total dose of LT4, μ/kg dose, tumor size, RIT (adjuvant activity), TSH, FT4, TG, and LM did not show correlation with QoL.

**TABLE 2:** Patients with differentiated thyroid carcinoma regarding the quality of life assessed by SF‐36 instrument.

SF‐36 domains	Differentiated thyroid carcinoma *n* = 51
Functional capacity	82.5 ± 18.1
Limitations due to physical aspects	71.9 ± 41.4
Pain	62.6 ± 25.2
General condition	50.1 ± 20.5
Vitality	53.3 ± 23.1
Social aspects	68.5 ± 27.4
Limitations due to emotional aspects	55.3 ± 42.3
Mental health	59.2 ± 21.9
Physical component summary	47.5 ± 8.3
Mental component summary	40.9 ± 12.5

*Note:* Variables are expressed as mean ± standard deviation.

### 3.2. BC

BC descriptive analysis in patients with DTC is given in Table 3.

**TABLE 3:** Descriptive analysis of body composition in patients with DTC.

	**Differentiated thyroid carcinoma *n* = 51**

Total body mass (kg)	77.3 ± 17.8
Lean mass (g)	45,144.4 ± 11,602.6
Total fat percentage (%)	38.3 ± 12.1
Fat mass (g)	30,782.7 ± 13,843.2
Fat mass index	11.3 ± 5.3
Baumgartner index	8.0 ± 1.6

*Note:* Variables are expressed as mean ± standard deviation. Baumgartner index: appendicular lean mass relative to body surface area.

**FIGURE 2:**
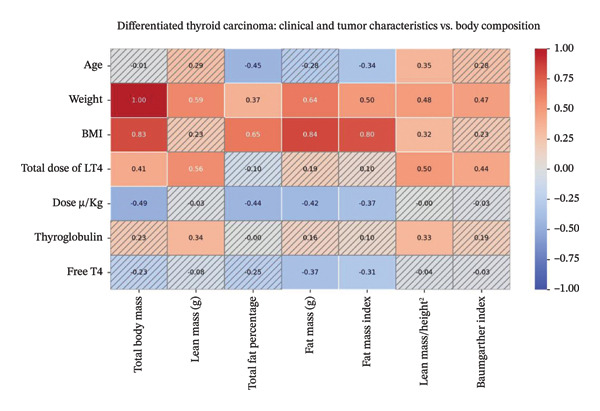
Spearman’s linear correlation heatmap demonstrates values of ῤ between demographic, anthropometric, clinical, laboratory, thyroid function, and tumor variables with body composition in patients with DTC. Values of *p* < 0.05 are not shaded.

Patients with DTC showed a negative correlation between age and TFP and FMI; between dose μg/kg and TBM, TFP, FM, and FMI; and between FT4 and FM and FMI. Positive correlation was found between age and total lean mass/height^2^; between weight and all BC assessments; between BMI and TBM, TFP, FM, FMI, and total lean mass/height^2^; between total LT4 dose and TBM, LM, FM, total lean mass/height^2^, and Baumgartner index; and between TG and LM and total lean mass/height^2^. TSH did not show correlation with the domains of BC (Figure 2, Supporting Material Table S4).

### 3.3. Relationship Between QoL and BC in Patients With DTC

**FIGURE 3:**
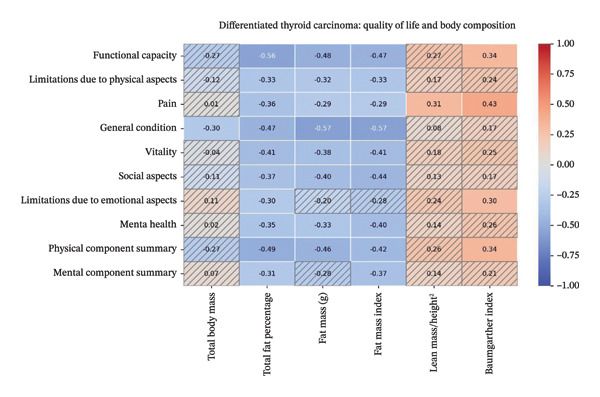
Spearman’s linear correlation heatmap demonstrates values of ῤ between the body composition variables and quality of life in patients with DTC. Values of *p* < 0.05 are not shaded.

In patients with DTC, there was a negative correlation between TBM and GC; between TFP and all domains; between FM and all domains except LE and MCS; and between FMI and all domains except LE. A positive correlation was found between total lean mass/height^2^ mass and pain and between Baumgartner index and FC, pain, LE, and PCS (Figure 3, Supporting Material Table S5).

### 3.4. Predictive Factors to BC

In patients with DTC, being female and higher FT4 and GC scores in SF‐36 were predictors of lower TBM. Higher age, LT4 dose, and GC score were predictors of lower FMI, and higher time of diagnosis was predictor of higher FMI. Higher age, LT4 dose, TSH, and PCS were predictors of lower TFP, and female sex and more diagnostic time were predictors of higher TFP. Higher age, LT4 dose, and GC score were predictors of lower FM (Table 4).

**TABLE 4:** Predictive factors related to body composition in differentiated thyroid carcinoma patients.

Variables	Total body mass		Fat mass index		Total fat percentage		Fat mass	
	Estimated parameter	*p* value	Estimated parameter	*p* value	Estimated parameter	*p* value	Estimated parameter	*p* value
Differentiated thyroid carcinoma								
Age (years)			−0.350	0.001	−0.372	< 0.001	−0.232	0.040
Sex (female *x* male)	−11.818	0.010	—	—	10.873	0.001	—	—
Free T4	−0.287	0.030	—	—	—	—	—	—
TSH					−0.252	0.0021	—	—
Levothyroxine dose mcg/kg	—	—	−0.327	0.004	−0.489	< 0.001	−0.312	0.009
Diagnostic time	—	—	0.298	0.008	0.213	0.007	—	—
Physical component summary	—	—	—	—	−0.247	0.001	—	—
General condition	−0.368	0.008	−0.484	< 0.001	—	—	−0.433	0.001

*Note:* Multiple linear regression analysis with selective stepwise variable selection was performed. R2 of the model = 0.3002; study variables included in the model of regression: age, time of diagnosis, free T4, TSH, TG thyroglobulin, functional capacity, GC general condition, mental health, PCS physical component summary and MCS mental component summary; variables not included in the model: body mass index and dose/mcg/Kg. O model was not changed with the inclusion of the SF36 components. R2 of the model = 0.5596.

## 4. Discussion

The present study demonstrated a relationship between BC changes and impaired QoL in patients with hypothyroidism after thyroidectomy, emphasizing the relevance of exploring this relationship in specific populations such as young patients with DTC. The findings of this study reinforce the clinical observation that even young patients with DTC, after undergoing TT, receiving adequate LT4 replacement with TH profile within RVs and an excellent treatment response, may still experience impaired QoL associated with alterations in BC. Our cohort was predominantly composed of young individuals (mean age: 35 years), almost 70% of them with normal or overweight, excluding menopause as a criterion, thereby minimizing common confounding factors that could harm BC and QoL. Consequently, the impairments observed cannot be attributed to age‐related changes but rather reflect the burden of DTC and its treatment. These results suggest that normalization of TH profile alone may not be sufficient to fully restore metabolic and functional homeostasis.

Hypothyroidism after TT has been consistently linked to weight gain [7]. TH plays a pivotal role in regulating energy metabolism, thermogenesis, lipolysis, and protein metabolism. TH receptors, particularly the TR‐β subtype, are highly expressed in the hypothalamus, where they modulate appetite, especially within the arcuate nucleus [8]. Moreover, the central action of T3 in the ventromedial hypothalamus involves AMPK activation, which reduces ceramide‐induced ER stress—stimulating thermogenesis in brown adipose tissue (BAT)—and activates JNK, thereby influencing hepatic lipid metabolism [9]. Through these mechanisms, T3 increases basal EE, enhances mitochondrial function, and regulates protein turnover, all of which are critical for maintaining healthy BC [10]. TH also modulates BAT activity and the browning of white adipose tissue, converting white adipocytes into cells with thermogenic capacity and thereby increasing EE [11].

Although the central role of TH in energy metabolism is established, its specific effects on LM, FM, and BMI remain debated. In our findings, higher FT4 and LT4 doses correlated with lower TBM, FM, TFP, and BMI, contrasting with prior studies that found no significant BC changes among hypothyroid patients on different LT4 regimens, despite mild TSH variation. Wang et al. [12] observed a reduction in FM after RIT without changes in LM, suggesting that hypothyroidism promotes fat accumulation while restoration of TH favors fat loss. However, TSH variations within the reference range seem not to significantly influence BC. Supporting this, Boeving [13] found no BC differences between hypothyroid patients with high‐normal versus low‐normal TSH.

Weight gain remains a common source of dissatisfaction and reduced QoL, despite appropriate LT4 replacement. Interestingly, some studies suggest that patients undergoing TT gain more weight than those with others hypothyroidism, even when biochemically euthyroid, implying that the absence of the thyroid gland itself may contribute to persistent alterations in metabolism and BC [14, 15]. In the literature, the groups most affected by hypothyroidism in terms of BC are predominantly women, symptomatic individuals, patients with more pronounced TSH elevation, and the elderly. However, our findings reinforce that even younger patients with THs within RVs may also present measurable impairments in BC, suggesting that the burden of permanent thyroid loss is not limited to traditionally high‐risk groups [16].

In our study, most participants were disease‐free or had minimal disease burden, but significant alterations in BC were still evident. These changes correlated consistently with lower QoL scores across several SF‐36 domains, indicating that the impact of DTC extends beyond oncologic outcomes. Even subtle changes in BC during early adulthood can meaningfully affect vitality, social functioning, and overall health perception. Excess FM was particularly detrimental, negatively influencing general health and physical function domains. Indeed, DTC survivors often report worse QoL than the general population, regardless of age or sex [17], with frequent use of medication for anxiety, pain, and depression [18]. Noto et al. identified predictors of anxiety and depression such as female sex, higher BMI, recurrent laryngeal nerve injury, and permanent hypoparathyroidism [19]. Other reports link cancer diagnosis and treatment duration to impaired QoL, independent of TSH or socioeconomic status [3, 20]. There are still few studies that have directly examined the interplay between BC and QoL in this population.

Importantly, hypothyroid patients on LT4 often report cognitive and mood complaints; however, consistent evidence linking these symptoms to biochemical parameters remains elusive. Samuel et al. described slightly lower FC scores in DTC patients on different LT4 doses, but overall differences were modest. Curiously, patients who perceived themselves as receiving higher LT4 doses reported better well‐being, despite having lower free T3 levels, raising questions about the role of T3 in QoL outcomes [13]. Most available studies focus on oncologic or biochemical endpoints, overlooking functional and psychosocial aspects.

Regarding LT4 therapy, our analysis showed a negative correlation between LT4 dose and FT4 with TBM, TFP, FM, and FMI. This likely reflects the use of higher LT4 doses to achieve lower TSH in high‐risk patients. However, excessive LT4 is not risk‐free, as it may induce arrhythmia, atrial fibrillation [21], dyslipidemia, cognitive decline, and insulin resistance, increasing cardiovascular risk [22]. To refine LT4 dosing, predictive models have been proposed, moving beyond weight‐based approaches. Samuel et al. [23] recommended using lean body mass (LBM), accurately measured by DXA, as a dosing guide to reduce overtreatment. This is especially relevant for older women with lower LBM, who are more vulnerable to iatrogenic thyrotoxicosis [24].

Altogether, our results confirm that even after TT and adequate LT4 replacement, DTC patients experienced impaired QoL, strongly related to BC alterations. Negative correlations were evident between BC and QoL, with TBM inversely associated with GC and TFP inversely associated with all SF‐36 domains. These data indicate that normalization of thyroid tests did not fully restore metabolic equilibrium or QoL in young athyreotic individuals. The absence of the thyroid gland, combined with lifelong LT4 dependence, seems to generate lasting metabolic and psychosocial effects. Thus, a multidisciplinary approach, including nutritional, physical, and psychological strategies, should be considered even for patients with excellent oncological prognosis.

A key limitation of our study is the lack of baseline BC data prior to thyroidectomy, preventing us from determining whether the higher FM and TFP observed were preexisting or developed after surgery and LT4 therapy. Lifestyle factors such as dietary habits and physical activity, as well as time since thyroidectomy, may influence BC and QoL outcomes. Although physical activity was assessed, dietary intake was not objectively measured, which represents a limitation and may have influenced our findings. Nevertheless, the robust association between adiposity measures and reduced QoL suggests that excess FM, regardless of origin, was a major determinant of impaired well‐being. The use of DXA, considered the gold standard for BC assessment, strengthens our findings beyond simple BMI‐based analysis. Prospective longitudinal studies with pre‐ and post‐thyroidectomy data are needed to establish causality. Limitations also include the absence of circulating T3 measurement, the lack of a control group, and no assessment of protein intake, which could affect LM preservation. Strengths of this work include the use of DXA and the evaluation of a homogeneous, young cohort, minimizing age‐related confounding.

In conclusion, young patients with DTC, even in the absence of advanced disease, showed impaired QoL associated with unfavorable BC changes, particularly increased FM, TFP, and FMI. From a clinical perspective, our findings highlighted the need to move beyond biochemical targets in the follow‐up of DTC patients. Even in young individuals with excellent oncological response and adequate TH replacement, significant impairments in BC and QoL may persist. This supports the implementation of a multidisciplinary approach involving endocrinologists, nutritionists, and exercise professionals, aiming not only at disease control but also at optimizing metabolic health and patient well‐being. Future prospective studies are warranted to clarify the causal pathways linking BC and QoL in this population.

## Funding

This work did not have financial support to be carried out.

## Disclosure

This study was presented partially in the 2025 American Thyroid Association Annual Meeting and published as an abstract (Thyroid, 35, Supplement S1, page e141, 2025. Ferrari C, Zantut‐Wittmann D. Higher body fat and poorer quality of life in differentiated thyroid carcinoma. DOI: 10.1177/10507256251378952).

## Conflicts of Interest

The authors declare no conflicts of interest.

## Supporting Information

Additional supporting information can be found online in the Supporting Information section.

## Supporting information


**Supporting Information** Supporting 1. Table S1: Descriptive analysis of histopathological characteristics, specific treatment, and evolution of patients with differentiated thyroid carcinoma. Supporting 2. Table S2: Descriptive analysis of patients with differentiated thyroid carcinoma in relation to lifestyle habits. Supporting 3. Table S3: Spearman’s linear correlation coefficients between demographic, anthropometric, and tumor characteristic variables with quality of life in patients with differentiated thyroid carcinoma. Supporting 4. Table S4: Spearman’s correlation coefficients between demographic, anthropometric, clinical, laboratory, thyroid function, tumor variables, and body composition in patients with differentiated thyroid carcinoma. Supporting 5. Table S5: Spearman’s correlation coefficients between body composition variables and quality of life in patients with differentiated thyroid carcinoma.

## Data Availability

The data that support the findings of this study are available from the corresponding author (DEZW) upon reasonable request.
