# Supplementary material for: Relationship Between Body Composition and Quality of Life in Young Patients With Differentiated Thyroid Carcinoma After Thyroidectomy
Source: Int J Endocrinol. 2026 May 22;2026:9268141. doi: 10.1155/ije/9268141 (PMC13195627; doi:10.1155/ije/9268141)
Supplement: Supplementary file 1 — Supporting Information Supporting 1. Table S1: Descriptive analysis of histopathological characteristics, specific treatment, and evolution of patients with differentiated thyroid carcinoma. Supporting 2. Table S2: Descriptive analysis of patients with differentiated thyroid carcinoma in relation to lifestyle habits. Supporting 3. Table S3: Spearman’s linear correlation coefficients between demographic, anthropometric, and tumor characteristic variables with quality of life in patients with differentiated thyroid carcinoma. Supporting 4. Table S4: Spearman’s correlation coefficients between demographic, anthropometric, clinical, laboratory, thyroid function, tumor variables, and body composition in patients with differentiated thyroid carcinoma. Supporting 5. Table S5: Spearman’s correlation coefficients between body composition variables and quality of life in patients with differentiated thyroid carcinoma. [file IJE-2026-9268141-s001.docx]

**SUPPLEMENTARY MATERIAL:**

**Table S1.** Descriptive analysis of histopathological characteristics, specific treatment and evolution of patients with differentiated thyroid carcinoma.

| Variable | | | Differentiated thyroid carcinoma (n=51) | | |
| --- | --- | --- | --- | --- | --- |
| Diagnostic time (years) | | | 7.0 ± 5.0 | | |
| Type of thyroid cancer | follicular | | 2 (3.9%) | | |
|  | papillary | | 49 (96.1%) | | |
| Size (cm) | | | 2.4 ± 1.8 | | |
| Focality | Multifocal | | 25 (50.0%) | | |
|  | Unifocal | | 25 (50.0%) | | |
| Encapsulated | | | 21 (47.7%) | | |
| Aggressive subtype of PTC | | | 10 (20.0%) | | |
| Extrathyroidal extension | | | 15 (33.3%) | | |
| Vascular invasion | | | 11 (26.2%) | | |
| Lymphatic invasion | | | 10 (22.7%) | | |
| Thyroidectomy | Total | | 35 (68.6%) | | |
|  | Total + neck dissection | | 16 (31.4%) | | |
| Radioiodine therapy (mCi, ablative dose) | | | 136.6 ± 34.4 | | |
| Radioiodine therapy (mCi, adjuvant or therapeutic dose) | | | 270.1 ± 137.7 | | |
| Radioiodine therapy (mCi, total dose) | | | 192.3 ± 140.1 | | |
| Tumor (T) | 1 | | 23 (48.9%) | | |
|  | 2 | | 7 (14.9%) | | |
|  | 3 | | 17 (36.2%) | | |
| Lymph node (N) | 0 | | 21 (44.7%) | | |
|  | 1 | | 26 (55.3%) | | |
| Metastases (M) | 0 | | 42 (89.4%) | | |
|  | 1 | | 5 (10.6%) | | |
| Initial Risk ATA | Low | | 16 (32.7%) | | |
|  | Intermediary | | 22 (44.9%) | | |
|  | High | | 11 (22.4%) | | |
| Response to treatment in the evaluation | Incomplete biochemistry | | 1 (2.0%) | | |
|  | Incomplete structural | | 9 (18.0%) | | |
|  | Excellent | | 26 (52.0%) | | |
|  | Undetermined | | 4 (28.0%) | | |
| Thyroglobulin (ng/mL) | | | | | 5.60 ± 25.05 |
| Anti-thyroglobulin antibody | Positive | 2 (4.2%) | | | |
|  | Negative | 46 (95.8%) | | | |
| Total dose of levothyroxine (µg) | | | | | 142.2 ± 45.87 |
| Dose of levothyroxine/ weight (µg/kg) | | | | 1.83 ± 0.49 | |

Variables expressed as mean + standard deviation; Variables expressed as median (minimum and maximum). ATA: America Thyroid Association.

**Table S2**. Descriptive analysis of patients with differentiated thyroid carcinoma in relation to lifestyle habits.

| Variable | | Differentiated thyroid carcinoma n=51 |
| --- | --- | --- |
| Physical activity | Active | 5 (9.8%) |
|  | Inactive | 46 (90.2%) |
| Smoking | Yes | 48 (94.1%) |
|  | No | 3 (5.9%) |
| Sun Exposure | Suitable | 24 (47.1%) |
|  | Inadequate | 27 (52.9%) |
| Caffeine | Bigger | 18 (35.3%) |
|  | Minor | 33 (64.7%) |

**Table S3**: Spearman's linear correlation coefficients between demographic, anthropometric and tumor characteristic variables with quality of life in patients with differentiated thyroid carcinoma.

| **Variables** | FC | LP | Pain | GC | Vitality | SA | LE | Mental Health | MCS | PCS |
| --- | --- | --- | --- | --- | --- | --- | --- | --- | --- | --- |
| **DIFFERENTIATED THYROID CARCINOMA (ῤ / p-value)** | | | | | | | | | | |
| Age | 0.30436  0.0316 | 0.30596  0.0307 | 0.23240  0.1044 | 0.16495  0.2523 | 0.27919  0.0496 | 0.43106  0.0018 | 0.38436  0.0059 | 0.43825  0.0015 | 0.45978  0.0008 | 0.21306  0.1374 |
| BMI | -0.28946  0.0437 | -0.16605  0.2542 | -0.09689  0.5078 | -0.41401  0.0031 | -0.12038  0.4100 | 0.18688  0.1985 | 0.01427  0.9225 | -0.07287  0.6188 | -0.02470  0.8662 | -0.32642  0.0221 |
| Diagnostic time | 0.32440  0.0215 | 0.35385  0.0117 | 0.20596  0.1513 | 0.30990  0.0285 | 0.13497  0.3500 | 0.24276  0.0894 | 0.14098  0.3288 | 0.12922  0.3711 | 0.13106  0.3643 | 0.37501  0.0073 |
| RIT ablative activity | 0.20302  0.1711 | 0.05049  0.7361 | 0.29136  0.0469 | 0.17716  0.2335 | -0.01770  0.9060 | 0.05436  0.7167 | 0.06047  0.6864 | -0.07790  0.6027 | -0.04163  0.7811 | 0.24978  0.0904 |
| RIT total activity | 0.25019  0.0899 | 0.15462  0.2994 | 0.32998  0.0235 | 0.24581  0.0958 | 0.08587  0.5660 | 0.16846  0.2577 | 0.021960  0.8835 | 0.04814  0.7480 | 0.00367  0.9805 | 0.36705  0.0112 |

BMI Body mass index, FC Functional capacity, LP Limitations due to physical aspects, GC General condition, SA Social aspects, LE Limitations due to emotional aspects. PCS Physical Component Summary; MCS Mental Component Summary. RIT Radioiodine therapy

**Table S4:** Spearman’s correlation coefficients between demographic, anthropometric, clinical, laboratory, thyroid function, tumor variables and body composition in patients with differentiated thyroid carcinoma.

| **Variables** | Total body mass | Lean mass (g) | TFP | Fat Mass (g) | FMI | Total lean mass / height² | Baumgartner Index |
| --- | --- | --- | --- | --- | --- | --- | --- |
|  | **DIFFERENTIATED THYROID CARCINOMA (ῤ / p-value)** | | | | | |  |
| Age | -0.009  0.948 | 0.287  0.052 | -0.452  0.001 | -0.284  0.055 | - 0.344  0.018 | 0.348  0.017 | 0.284  0.055 |
| Weight | 0.996  <0.000 | 0.585  <0.000 | 0.370  0.011 | 0.642  <0.000 | 0.498  0.000 | 0.484  0.000 | 0.470  0.001 |
| BMI | 0.826  <0.000 | 0.232  0.119 | 0.653  <0.000 | 0.841  <0.000 | 0.798  <0.000 | 0.320  0.030 | 0.231  0.122 |
| Total dose of LT4 | 0.412  0.004 | 0.556  <0.000 | -0.099  0.509 | 0.193  0.198 | 0.099  0.511 | 0.496  0.000 | 0.436  0.002 |
| Dose µ/kg | -0.491  0.000 | -0.029  0.843 | -0.440  0.002 | -0.422  0.003 | -0.370  0.011 | -0.000  0.997 | -0.032  0.831 |
| Free T4 | -0.322  0.028 | -0.030  0.840 | -0.328  0.025 | -0.337  0.021 | -0.259  0.081 | -0.020  0.892 | 0.008  0.957 |
| Thyroglobulin | 0.248  0.107 | 0.363  0.016 | -0.026  0.865 | 0.139  0.372 | 0.081  0.601 | 0.337  0.026 | 0.212  0.170 |

BMI body mass índex; TFP total fat percentage, FMI fat mass índex, LT4 levothyroxine.

**Table S5:** Spearman’s correlation coefficients between body composition variables and quality of life in patients with differentiated thyroid carcinoma.

| **Variables** | FC | LF | Pain | GC | Vitality | SA | LE | Mental Health | MCS | PCS |
| --- | --- | --- | --- | --- | --- | --- | --- | --- | --- | --- |
|  | DIFFERENTIATED THYROID CARCINOMA (ῤ / p-value) | | | | | | | | | |
| Total body Mass | -0.273  0.066 | -0.118  0.433 | 0.013  0.931 | -0.298  0.043 | -0.036  0.811 | -0.11  0.455 | 0.113  0.452 | 0.020  0.892 | 0.070  0.643 | -0.271  0.067 |
| Total Fat Percentage | -0.556  <0.000 | -0.325  0.027 | -0.361  0.013 | -0.465  0.001 | 0.414  0.004 | -0.37  0.010 | -0.298  0.043 | -0.346  0.018 | -0.312  0.034 | -0.493  0.000 |
| Fat Mass | -0.477  0.000 | -0.317  0.031 | -0.293  0.048 | -0.572  <0.000 | 0.376  0.009 | -0.40  0.005 | -0.197  0.188 | -0.326  0.026 | -0.282  0.056 | -0.462  0.001 |
| Fat Mass index | -0.472  0.000 | -0.327  0.026 | -0.293  0.047 | -0.565  <0.000 | -0.412  0.004 | -0.43  0.002 | -0.283  0.056 | -0.403  0.005 | -0.370  0.011 | -0.424  0.003 |
| Total lean mass / height ² | 0.268  0.071 | 0.169  0.260 | 0.314  0.033 | 0.075  0.617 | 0.176  0.239 | 0.125  0.407 | 0.236  0.113 | 0.137  0.361 | 0.137  0.360 | 0.258  0.082 |
| Baumgartner Index | 0.335  0.022 | 0.242  0.104 | 0.428  0.003 | 0.169  0.261 | 0.248  0.096 | 0.168  0.264 | 0.302  0.040 | 0.255  0.086 | 0.337  0.022 | 0.214  0.152 |

FC Functional capacity, LF Limitations due to physical aspects, GC General condition, SA Social aspects, LE Limitations due to emotional aspects. PCS Physical Component Summary; MCS Mental Component Summary; GC General condition, PCS Physical Component Summary.
